# Identification and Methods of Influencing the Oxidation States of Mn and Ce in Silicate Glasses

**DOI:** 10.3390/ma18091948

**Published:** 2025-04-25

**Authors:** Jakub Volf, Petr Vařák, Maksym Buryi, Martin Kormunda, Pavla Nekvindová

**Affiliations:** 1 Department of Inorganic Chemistry, University of Chemistry and Technology, Technická 5, 166 28 Prague, Czech Republic; volfa@vscht.cz (J.V.); varakp@vscht.cz (P.V.); 2 Institute of Plasma Physics, Czech Academy of Sciences, U Slovanky 2525/1a, 182 00 Prague, Czech Republic; buryi@ipp.cas.cz; 3 Department of Physics, Faculty of Science, Jan Evangelista Purkyně University in Ústí nad Labem, Pasteurova 3544/1, 400 96 Ústí nad Labem, Czech Republic; martin.kormunda@ujep.cz

**Keywords:** photoluminescence, borosilicate glass, cerium, manganese, oxidation states, optical basicity, EPR, XPS

## Abstract

Non-hygroscopic borosilicate glasses containing Ce^3+^ and Mn^2+^ ions were prepared using the conventional melt-quenching method. The electrochemical equilibrium of the Ce and Mn oxidation states has a significant effect on the energy levels and luminescence of both elements. Consequently, the oxidation states in the glasses were analyzed using a combination of XPS, EPR, and absorption spectroscopy. The oxidation–reduction equilibrium was altered by systematically changing three factors: the Mn concentration, the presence or absence of SnO as a reducing agent, and the optical basicity of the glass. Upon excitation with light with a wavelength of 320 nm, the prepared glasses exhibited a blue luminescence band in the region of 350–450 nm, corresponding to the Ce^3+^ ion, and a broad, weak red luminescence emission in the region of 540–640 nm, corresponding to Mn^2+^ ions. To obtain a high luminescence intensity for both bands, it was necessary to reduce the MnO content below 1 mol.%. Furthermore, doping the glasses with Sn^2+^ ions helped to maintain both cerium and manganese in low oxidation states, resulting in measurable luminescence in both observed bands. These low oxidation states of Ce and Mn can also be achieved by reducing the optical basicity of the glass through the addition of MgO. The general relationships obtained could potentially be applied in the production of light-emitting diodes or field-emission displays that utilize energy transfer.

## 1. Introduction

Cerium-doped materials have garnered significant attention due to their potential as tunable phosphors and scintillators operating in the broad UV and VIS emission regions. Cerium has been incorporated into a wide range of crystalline materials. Among them, silicate-based phosphors stand out for their high quantum efficiency, excellent color purity, remarkable chemical and thermal stability, extended persistence, ability to produce multi-color emissions, etc. [1,2,3]. The direct doping of amorphous materials, such as glass-ceramics or glasses, with Ce is also being intensively studied. This approach offers the advantage of enabling the preparation of thermally stable optical fibers [4,5]. Cerium exists as a solid in its stable oxidation states (+III and +IV). The high emission intensity and broad emission band arise from the 4f–5d spin-allowed transition and the phonon band of the Ce^3+^ ion. Moreover, the emission spectra of Ce^3+^ can overlap with the excitation spectra of other ions, such as Mn^2+^, Tb^3+^, Dy^3+^, and Eu^2+^, which enables energy transfer from Ce^3+^ to these ions when they are co-doped into suitable matrices. Consequently, these systems can generate tunable emission colors [6,7,8,9,10].

One of the mostly studied sensitizer–activator pairs is Ce^3+^ sensitizing Mn^2+^. This pair typically exhibits luminescence in both the blue and red regions of the visible spectrum, with the precise position of the bands depending on the glass matrix. However, due to the high variability in glass compositions, a significant challenge lies in the fact that both elements have multiple oxidation states, which can be strongly affected by the glass matrix and quite difficult to identify. Although both elements have characteristic absorption and luminescence bands, these are simultaneously dependent on their oxidation states and the matrix structure. Cerium occurs in Ce^3+^ and Ce^4+^ oxidation states, but only the former exhibits luminescence in the region around 350 nm [11]. The relative quantities of these states in glass can be partially determined using X-ray photoelectron spectroscopy (XPS) with spectrum deconvolution. Cerium peaks have been observed between 875 eV and 925 eV, with the peak at 915 eV indicating the presence of Ce^4+^ [12,13,14]. However, to have an understanding of the mechanisms of electron transitions in glass, it is useful to determine the Ce^3+^/Ce^4+^ ratio accurately and to gain insights into the structural role of cerium in glass. For this purpose, XAS (X-ray absorption spectroscopy) methods in combination with XANES (X-ray absorption near-edge structure) are nowadays the preferred methods [15,16]. Manganese occurs in several oxidation states, with Mn^2+^ and Mn^3+^ being the most common in glasses [17,18,19,20,21,22]. However, higher oxidation states, specifically Mn^4+^ and Mn^5+^, have also been reported [23,24]. Absorption spectroscopy can be employed to partially detect manganese oxidation states. The valence of manganese can be confirmed using electron paramagnetic resonance (EPR). Mn^2+^ has a characteristic EPR signal with a hyperfine structure, whereas Mn^3+^ does not appear in the EPR spectrum [24,25,26]. Furthermore, Mn^4+^ can be identified and distinguished from Mn^2+^ using the EPR method [27,28]. However, quantification remains challenging.

Concerning the luminescence of the Ce^3+^–Mn^2+^ pair in amorphous materials, the oxidation states of the ions and their immediate structural vicinity play a crucial role in the photoluminescence mechanisms. Therefore, various glass matrices have been studied in recent years. Studies on doped-phosphate and zinc-phosphate glasses have revealed intense luminescence of Ce^3+^ around 350 nm and broad luminescence of Mn^2+^ around 580 nm [29,30,31,32]. These materials have been proposed for application in LEDs. The sensitizer–activator pair Ce^3+^–Mn^2+^ has also been observed in silicate, borate, borosilicate, fluorosilicate, and high-transmission glasses [33,34,35,36,37]. The composition of the glass is generally influenced by the vicinity of ions, which favor one oxidation state over another. One method to estimate the effect of composition on the valence of ions is by detecting their optical basicity [38]. In glasses, which are systems characterized by remarkable variability in composition and internal structure, it is crucial to accurately determine the concentrations of individual ions, their oxidation states, and the crystal vicinity of the phosphor to understand fully the mechanism of luminescence. Consequently, this paper focuses on exploring methods to determine or influence the oxidation states of cerium and manganese, as well as their oxidation–reduction equilibrium. For this purpose, the Ce^3+^–Mn^2+^ ion (sensitizer–activator) pair was doped into stable, non-hygroscopic borosilicate glasses prepared using the conventional melt-quenching method. The effect of the ratio of the two ions and the changes in their oxidation states on photoluminescence was investigated. The influence of the glass structure on the properties and luminescence of both ions was demonstrated through systematically changing the optical basicity of the glasses. The prepared glasses and the derived general relationships have potential applications in light-emitting diodes (LEDs) and field-emission displays (FEDs) that utilize energy transfer (ET).

## 2. Materials and Methods

### 2.1. Glass Preparation

To study the influence of the glass composition and the host-matrix structure on the photoluminescence properties of Ce and Mn ions, four groups of glass matrices were prepared. The general composition of the glass was SiO_2_–B_2_O_3_–Al_2_O_3_–Na_2_O–MeO, where Me = Mg, Ca, or Ba. The Ce content was kept constant at 1 mol.% CeO_2_ while the Mn content was changed. The first set of glasses contained 2 mol.% MnO, the second contained 1 mol.% and the third contained 0.2 mol.%. Within these individual sets, the content of the elements affecting basicity was adjusted such that the glasses in each set contained the same amount of Mg, Ca, or Ba. In addition, the glasses with 1 mol.% MnO were also prepared with 1 mol.% SnO, a reducing agent. Finally, reference glasses containing Ba and Ce (Ba–Ce standard) and Ba and Mn (Ba–Mn standard) were produced. The compositions of the glasses are summarized in Table 1. Cerium, manganese, and tin were substituted for silicon in the glass structure, i.e., one atom of manganese replaced one atom of silicon as a network former.

The various types of glass were prepared using a standard melt-quenching technique. The chemicals employed for the glass preparation included SiO_2_ (Sigma Aldrich, St. Louis, MO, USA, high-purity grade), H_3_BO_3_ (Penta, Prague, Czech Republic, p.a.), Al(OH)_3_ (Lach-Ner, Neratovice, Czech Republic, p.a.), MgO (Penta, pure), Na_2_CO_3_ (Penta, pure), CaCO_3_ (Penta, p.a.), BaCO_3_ (Spolana, Neratovice, Czech Republic, pure), MnO (Sigma Aldrich, 99%), CeO_2_ (Sigma Aldrich, 99.9%). The components were weighed to produce 50 g of the final bulk glass, mixed, and placed into a platinum crucible. The glass was melted at 1400–1500 °C for 4.5 h from the end of loading, followed by rapid quenching in water. For the removal of inner mechanical tension, the glass was annealed at the onset of the softening zone for 5 h. The prepared bulk glass was cut into samples with dimensions of approximately 20 × 10 × 1 mm, which were polished to optical quality using a suspension of cerium dioxide. This yielded uniform, transparent glass samples without visible defects such as phase separation, crystallization, or cracks.

### 2.2. Glass Characterization

The XRF measurements of the glass composition were conducted using a Thermo ARL Performix XRF spectrometer (Waltham, MA, USA) equipped with an Rh lamp, or the Axios instrument from PANalytical (Malvern, UK), which employs the wave-dispersion principle to perform measurements. The output of the analysis was the composition of the glass in the form of oxides. This analysis determined the content of all periodic-table elements except for light elements (H, He, Li, Be, B, and C) as well as the total amount of the elements. For our glasses, the original amount of B_2_O_3_ was thus added to the calculation of the composition based on XRF signals for all the other elements. The absolute error of this method did not exceed 0.1 wt. %.

The glass density was measured using a pycnometer, and the average of five measurements was recorded. The resulting glass dispersion curve was fitted to the Cauchy equation [37].

The refractive index of the glass was measured using a Metricon prism coupler at five different wavelengths (473, 633, 964, 1311, and 1553 nm). The final values were obtained by averaging the measurements from five samples. The resulting glass dispersion curve was fitted to the Cauchy equation [39]:

(1) $$n=A+\frac{B}{\lambda^{2}}+\frac{C}{\lambda^{4}}\;,$$

where n is the refractive index, λ is the wavelength, and A, B, and C are fitted parameters.

The structure of the glasses, especially the oxidation states of Mn, was examined using the electron-paramagnetic-resonance (EPR) method and a Bruker ELEXSYS E580 spectrometer (Billerica, MA, USA) in the X-band at a frequency of 9.4 GHz at room temperature. The sensitivity of the instrument is approximately 1012 spins/mT. The magnetic induction values were in the range of 0–7000 G.

Another method to determine the composition—and especially the oxidation state—of cerium is X-ray photoelectron spectroscopy (XPS). XPS spectra were obtained using a Phoibos 100 X-ray photoelectron spectrometer (SPECS) (Hertfordshire, UK) operating in the fixed-analyzer-transmission (FAT) mode with a five-channel MCD-5 detector (SPECS). A monochromatized X-ray source XR50 with FOCUS 500 (SPECS) was employed, featuring an Al X-ray tube and a Ka line (energy of 1486.6 eV) at 15 kV and 400 W; a flood gun FG 20 was utilized at 1 eV 100 µA. The sample was mounted on double-sided carbon conductive adhesive tape affixed to a stainless-steel sample holder. The survey spectrum was acquired at a pass energy of 40 eV, whereas high-resolution spectra were recorded at a pass energy of 10 eV or 40 eV as needed. The spectral analysis was conducted in CasaXPS, employing the Shirley background model.

The absorption spectra were measured in the range of 250–1000 nm using an AvaSpec StarLine spectrometer (Apeldoorn, The Netherlands) in a through-hole arrangement, with a step size of 0.4 nm. For each sample, 200 spectra were averaged with an integration time of 45 ms. Deuterium and halogen lamps were simultaneously employed as light sources. The reflective part of the attenuation was calculated from the interpolated values of n with the assumption that the incident ray was perpendicular to the parallel surfaces of the glass sample. Subsequently, the absorption and extinction coefficients were calculated. Since the trends in the dependence of both coefficients are identical, only the extinction-coefficient spectra are presented here.

The luminescence of the glasses was measured in a reflection arrangement using two setups. The excitation–emission maps were obtained using a Cary Eclipse fluorescence spectrophotometer from Agilent Technologies, Inc. (Santa Clara, CA, USA), equipped with a xenon lamp as a light source. The measuring range of the setup was 200–900 nm for both emission and excitation wavelengths. The slit width of the excitation monochromator was set to 2.5 nm, while that of the emission monochromator was set to 20 nm. The spectra were processed in OriginPro 2019b, with Rayleigh scattering bands. More accurate measurements at selected excitation wavelengths were performed using a Fluorolog^®^-3 Extreme spectrometer from Horiba (Kyoto, Japan). A xenon lamp was used as the light source. The emission spectra were recorded in the range of 330–900 nm and evaluated using OriginPro.

### 2.3. Optical-Basicity Evaluation

Optical basicity was chosen as a means to quantify the basicity of the glass matrices. The theoretical values were calculated using the following formula:

(2) $$\Lambda_{th}=\frac{\sum x_{i}N_{i}^{O}\Lambda_{i}}{\sum x_{i}N_{i}^{O}},$$

where Λ_th_ represents the optical basicities of the oxides, x_i_ refers to the molar ratios of these oxides, and $N_{i}^{O}$ denotes the number of oxide atoms in the oxide molecules. The optical basicities of the oxides were obtained from [40,41,42]. Alternatively, the optical basicity could be determined experimentally based on refractive-index, density, and cation polarizability measurements. According to [41], the derivation involves the following equations:

(3) $$\Lambda =1.67\left(1-\frac{1}{\alpha_{0}}\right)=1.67\left(1-\frac{1}{\alpha_{m}-\sum \frac{\alpha_{c}.x_{c}}{x_{0}}}\right)=1.67\left(1-\frac{1}{\frac{3R_{m}}{4\pi N_{A}}-\sum \frac{\alpha_{c}.x_{c}}{x_{0}}}\right),$$

where Λ is the optical basicity of the glass, α_0_ is the polarizability of the oxygen ion in the glass, x_0_ is the molar ratio of oxygen, α_m_ is the molar polarizability of the glass, α_c_ is the polarizabilities of the cations, x_c_ is their molar ratios, and R_m_ is the molar reflectivity calculated from the refractive-index values using the following formula:

(4) $$R_{m}=\frac{M}{\rho }\cdot \frac{n^{2}-1}{n^{2}+2}.$$

The refractive index of the glass was measured, and the n value at the wavelength of 589 nm was calculated based on the disperse curves. The density was determined pycnometrically, and the polarizability values were taken from [40,43].

## 3. Results

### 3.1. General Properties of the Prepared Glass

The prepared samples were first characterized based on their basic properties. The compositions of the glasses were verified using XRF. The concentrations of the individual oxides obtained from these measurements are presented in Table 1.

Subsequently, the density of the prepared glasses was determined pycnometrically, and the refractive index was measured. The measured value at 632 nm is provided in Table 2, along with the dispersion of the glasses, characterized by the parameters A, B, and C. The characteristic values for individual glasses are summarized in Table 2.

The dispersion curve was used to calculate the refractive-index value at a wavelength of 589 nm. Based on this value, the theoretical and determined basicity values of the glasses were computed according to formulae 2, 3, and 4. Table 2 provides a comparison of these two optical-basicity values.

**Figure 1:**
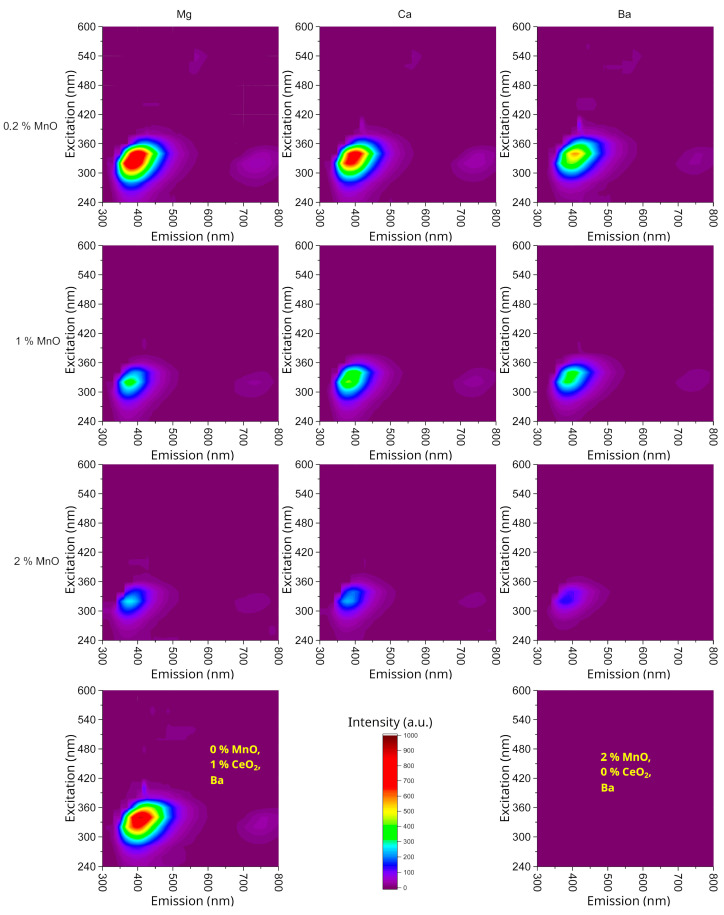
The excitation–emission maps of the glasses, sorted into columns based on divalent ions and into rows based on manganese concentration.

Figure 1 presents the results of the excitation–emission mapping, which illustrates the luminescence of the prepared samples. The excitation wavelength was varied with a step of 20 nm in the range of 240–600 nm, whereas the emission spectra were measured in the wavelength range of 300–800 nm with a step of 1 nm. In the spectra, the excitation wavelength is plotted on the vertical axis, the corresponding emission wavelength is plotted on the horizontal axis, and the third dimension of the figure represents the intensity of the measured emission, which is expressed using a color scale. The individual rows display the changes in the spectra caused by the alkaline-earth metal, whereas the columns show the effect of increasing the manganese content.

It is evident from the results in Table 2 that an increase in the mass and radius of the alkaline-earth metal (Mg → Ca → Ba) led to an increase in both the density and the refractive-index values of the prepared glasses, as expected. An increase in the concentration of manganese as well as tin resulted in a rise in both of the above-mentioned values, although the change was more moderate.

The theoretical value of the optical basicity (Λ_th_) increases in the following order: Mg → Ca → Ba. When we compare glasses with the same divalent cation, e.g., Ba glass, but with different Mn concentrations, the differences were very small. The effect of adding tin was negligible as well. Compared with the theoretical values, the experimentally determined values of optical basicity (Λ) generally provide a more accurate reflection of the glass composition because they take into account the real properties of the glass—such as the density and refractive index—and include the exact oxidation states of cerium and manganese. In general, the optical-basicity values clearly indicated that the glasses containing barium and calcium exhibited comparable basicity (with slight differences observed), whereas magnesium-containing glasses had significantly lower values.

The basic excitation–emission maps in Figure 1 clearly show Ce^3+^ luminescence with excitation at 325 nm and emission around 385 nm, corresponding to the transitions 5d → ^2^F_5/2_ and 5d → ^2^F_7/2_ [44]. In the standard sample with a 0% Ce content, no luminescence was observed, whereas in the sample with zero manganese content, the Ce^3+^ luminescence was very intense. The presence of manganese in the glass apparently suppresses the Ce^3+^ luminescence. Concerning the effects of Ba, Ca, and Mg, the intensity of the Ce^3+^ luminescence generally exhibited an increasing trend in this type of glass. Manganese-luminescence transitions with an emission around 600 nm were not visible.

Based on these maps, further measurements were conducted using a higher-resolution spectrometer with a suitable excitation wavelength, namely 320 nm. At the same time, the oxidation states of Mn and Ce were determined through the appropriate analytical methods, and the effects of glass basicity as a structural change on these oxidation states of the luminescent elements were compared.

### 3.2. The Determination of the Oxidation States of Ce and Mn

**Figure 2:**
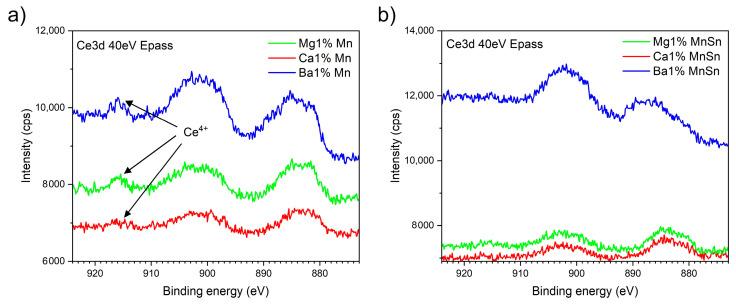
The XPS spectra of the glasses containing 1 mol.% MnO: (**a**) without SnO addition; (**b**) with SnO addition.

XPS analysis was used as the baseline method for determining the oxidation states of mainly the cerium and manganese. In general, it can also be applied to simpler oxide glasses to detect the ratio of bridged to non-bridged oxides, providing insights into the interconnectedness of the glass network. In our experiment, we analyzed two sets of glass triplets (Mg, Ca, and Ba): one containing 1 mol.% MnO (labeled Me1%Mn) and another with the same Mn concentration but with the addition of SnO (labeled Me1%MnSn). Figure 2 shows a comparison of the bands in the energy region of 875–920 eV of Ce3d, primarily reflecting the oxidation states of cerium. The band at 916 eV is attributed exclusively to Ce^4+^ [13], whereas the other two visible bands consist of multiple peaks corresponding to both Ce^3+^ and Ce^4+^, along with overlapping Mn LMM and Ba MNN Auger peaks. A comparison of Figure 2a,b reveals that SnO-containing glasses had a lower intensity band at 915 eV (in the case of the glass with Ba, the band is undetectable), which confirms the reduction of Ce^4+^ to Ce^3+^.

The energy bands of manganese typically appear around 640 eV in the XPS spectrum [45]. However, they were not visible in the depicted spectra because the Mn content was too low to determine the oxidation state, primarily due to the significant difference in RSF—13.91 for Mn2p and 51.62 for Ce3d—despite similar concentrations in the source material (see Table 1). In addition, the measured spectra contained a band around 530 eV, composed of the peaks of bridging oxygen atoms (BOs) and non-bridging oxygen atoms (NBOs) [46]. Nevertheless, these two bands could not be identified, probably because the high number of elements in the glass formed multiple types of bonds with oxygen, oscillating between covalent BOs and ionic NBOs.

**Figure 3:**
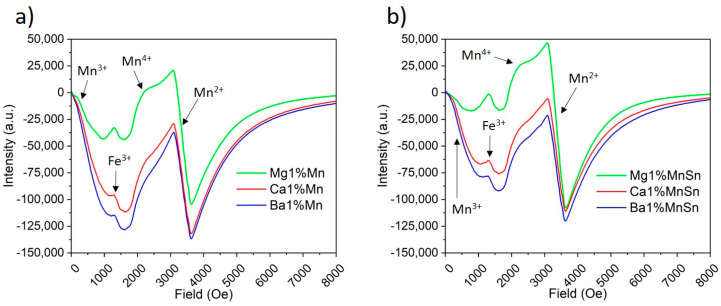
The EPR spectra of the glasses containing 1 mol.% MnO: (**a**) without SnO addition; (**b**) with SnO addition.

Since the XPS method provided practically no information on the oxidation states of manganese, we utilized the EPR method. We analyzed the same two sample triplets as in the previous case (1 mol.% MnO with and without SnO). Figure 3 shows a line at B = 3355 G (corresponding to a g-factor (g) ≈ 2.00), which is typical of spherical Mn^2+^ ions [47]. The second line, around B = 2350 G (g ≈ 2.86), which overlapped with the Mn^2+^ resonances, was attributed to Mn^4+^ at low-symmetry sites [48]. Finally, the line around B = 1450 G (g ≈ 4.6) is close to g ≈ 4.3, which is often observed for rhombic Fe^3+^ [25].

Assuming a Lorentzian shape of the resonance lines, their relative intensities were calculated as a product of the peak-to-peak signal intensity and the square of the peak-to-peak width. The intensity of the dominant line corresponding to Mn^2+^ increases in the Ba → Ca → Mg series was significantly higher in the magnesium-containing glasses. Furthermore, this line was affected by the addition of SnO, resulting in a higher intensity in all the glasses than in the tin-free samples. In contrast, the intensity of the other lines decreased with SnO doping. This implies a higher Mn^2+^ content in the Mg-containing and SnO-doped glasses at the expense of other oxidation states.

There was another resonance line detectable below 1000 G (Figure 3), which was likely produced by Mn^3+^. Logically, when Mn^2+^ and Mn^4+^ are present, there should be a non-zero probability of Mn^3+^ existing as well unless specific and charge compensation is introduced (which is not the case in this work). Since Mn^3+^ is a non-Kramers ion, one may expect large fine splitting, resulting in the spectral localization of the line below 1000 G at the X-band (see the Experimental section). Its intensity was approximately the same in the Ca- and Ba-based glasses, whereas it was about 3–4 times weaker in the Mg-containing glass, regardless of whether Sn was present or not. This can be explained by the fact that Mg has the smallest ionic radius among the three samples (Mg (approximately 0.6 Å on average when considering all possible oxygen coordination numbers) vs. Ca (about 1 Å) and Ba (ca 1.5 Å)). The average ionic radius of Mn is slightly below 0.6 Å. The values of the radii were obtained from [49]. Given that glass is a less rigid system than a single crystal, the size of the replacing ion can be expected to play a significant role in effective substitution.

### 3.3. The Absorption of the Prepared Glasses

**Figure 4:**
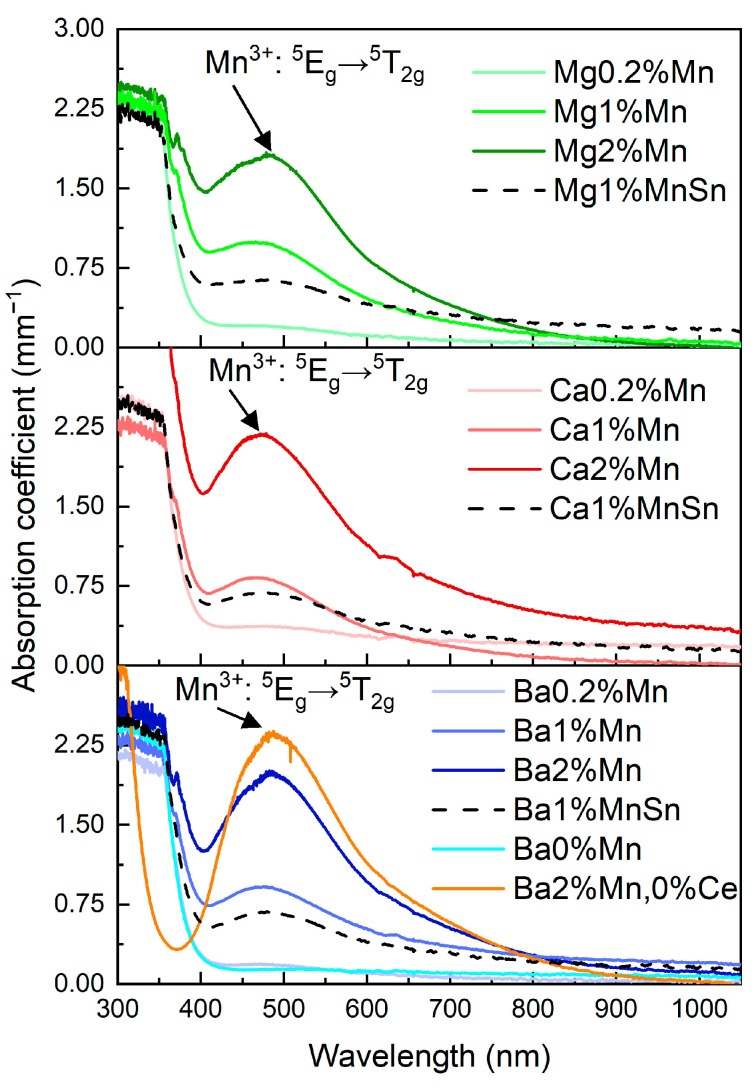
The absorption spectra of the Ce- and Mn-doped glasses: the influence of the alkaline-earth metal (in the column), the influence of the MnO content (the individual parts of the figure), and the influence of the SnO content (black dashed curves).

Figure 4 illustrates the impact of increasing the manganese content on the shape of the absorption spectra. Simultaneously, it demonstrates how different types of alkaline-earth metals influence the spectra. The Ce and Mn reference glasses are shown for the Ba-containing glass. It is evident from the figure that the Ba–Ce reference glass doped only with Ce exhibited no significant absorption in the UV–VIS region except for the absorption edge. In the spectra of the Ba–Mn reference glasses, the peak around 480 nm, attributed to the ^5^E_g_ → ^5^T_2g_ transition of the Mn^3+^ ion, is clearly visible, confirming the partial presence of Mn^3+^ in the glass. The intensity of the Mn^3+^ band increased with the Mn concentration. Its asymmetry is probably associated with the Jahn–Teller effect and lower site symmetry [24,33].

Looking at the spectra, a clear red shift in the Ce/Mn co-doped glasses compared with the Ce-doped glass (yellow curve) was obvious. This shift was clearly due to the presence of Ce in the glass structure [50,51]. Cerium in glasses shows a yellow coloration, and the absorption edge red shift corresponds to the spin-allowed 4f-5d transition of Ce^3+^ [30]. Moreover, in the spectra of the Mg2%Mn and Ba2%Mn glasses, a small peak at 371 nm was observed. The origin of this peak remains unclear. According to [21,50], this could be a Mn^2+^ transition (^6^A1g(^6^S) − ^4^T2g(^4^D)) related to the presence of cerium in the glass. On the other hand, this peak could also be related to the changes in the Ce^3+^/Ce^4+^ ratio in the glass because (a) this peak is absent in the spectrum of the Ba-Mn reference glass without Ce, (b) its intensity increases with the manganese concentration in the glass, and (c) the band completely disappears with the addition of tin to the glass.

Figure 4 further demonstrates the relatively minor influence of the different alkaline-earth metal oxides on the shape and intensity of the spectra. The extinction-coefficient values of glasses with the same Mn concentration were similar. The black dashed curves in Figure 4 illustrate the effect of tin addition on the shape and intensity of the spectrum. It compares glasses containing 1.0 mol.% MnO (solid line) with those containing 1.0 mol.% MnO and 1.0 mol.% SnO (dashed line). It is evident that the addition of SnO reduced the intensity of the absorption at 480 nm, which is associated with the Mn^3+^ ion, in all three types of prepared glasses. Moreover, there was a red shift of the maximum peak. This is consistent with the assumption that manganese (+III) ions are reduced to lower oxidation states in the presence of Sn^2+^ ions and is in good agreement with the lighter coloration of the glasses.

Following the method from [24,52,53], a deconvolution of the absorption spectra was performed. The bands at 500 nm (20,000 cm^−1^) and 469 nm (21,300 cm^−1^), corresponding to Mn^3+^ and Mn^4+^, respectively, were simulated. The band asymmetry observed mainly in the Ba–Mn standard glass can be explained, according to [24], by the band at 680 nm (14,700 cm^−1^), corresponding to Mn^5+^. Nevertheless, when that band was considered in the deconvolution, the results were not satisfactory. Therefore, the band was not taken into account and we assumed that the band asymmetry arose from another effect. The results are summarized in Table 3, from which the following trends are evident: (a) the manganese content of the glasses increased the content of both bands; (b) increasing the basicity (Mg → Ca → Ba order) did not cause significant changes in the content of the individual deconvoluted bands; and (c) when SnO was added to the glasses, the ratio of the bands at 500 and 469 nm changed significantly, corresponding to the expected reduction of Mn^4+^ to lower oxidation states.

### 3.4. The Luminescence of the Prepared Glasses

**Figure 5:**
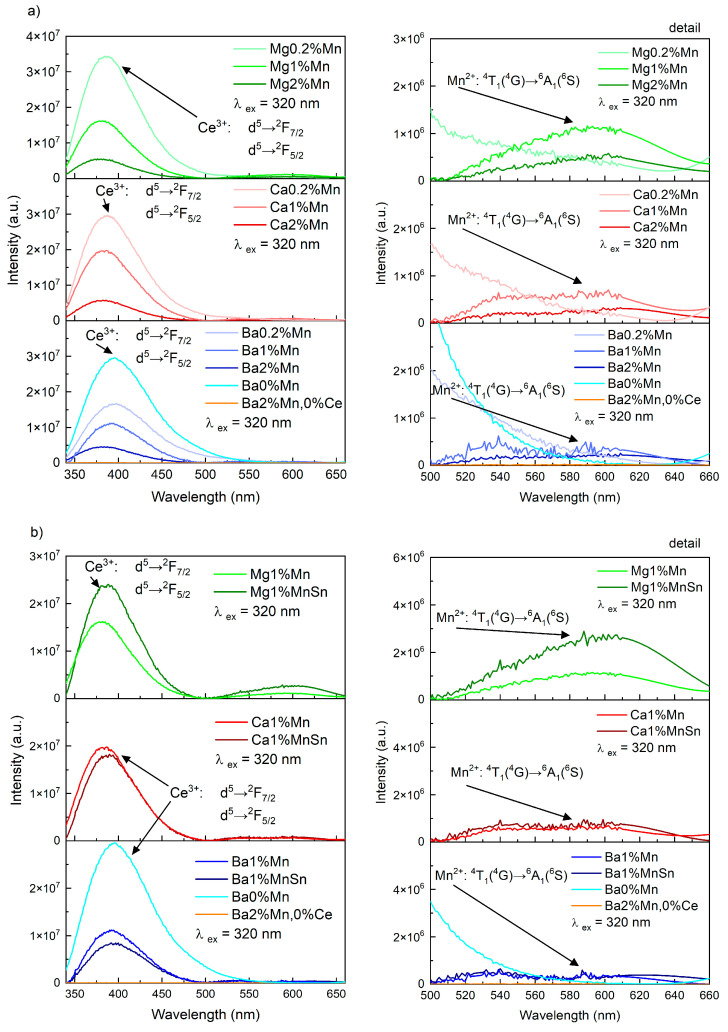
The luminescence spectra of the Ce- and Mn-doped glasses: (**a**) the influence of the alkaline-earth metal (in the column) and the influence of the MnO content (the individual parts of the figure); (**b**) the influence of the SnO content.

Luminescence spectra, measured with better resolution and excited at 320 nm, are presented in Figure 5a. The full spectra are shown on the left, while the detailed region in the range of 500–660 nm is displayed on the right.

As in the excitation–emission maps, the band corresponding to the ^5^d→^2^F_7/2_ and ^5^d→^2^F_5/2_ transitions of the Ce^3+^ ion is visible in the range of 350–450 nm [44]. In the detailed view of the figure on the right, it is possible to observe the luminescence at 600 nm, which was attributed to the ^4^T_1_(^4^G) → ^6^A_1_(^6^S) transition of the octahedrally coordinated Mn^2+^ ion [21,54]. Its intensity is usually more than an order of magnitude lower than that of the Ce^3+^ luminescence. The highest intensity for this band was observed in the glasses containing Mg and the lowest intensity was observed in those with Ba.

Figure 5b illustrates the effect of SnO addition on the measured spectra in glasses containing 1 mol.% MnO. It is evident that tin affected both the position and intensity of the two observed bands. The position of the band corresponding to Ce^3+^ shifted to higher wavelengths with the addition of SnO, which can be attributed to the splitting of d-orbitals resulting from the increased optical basicity. The intensity of the emission increased, with the highest intensity increase observed in the Mg-containing glasses. In these glasses, the addition of tin also significantly affected the band corresponding to manganese emission—the intensity was 2.4 times stronger and its maximum shifted from 594 to 602 nm. In the other types of glass, i.e., the barium and calcium glasses, this effect of tin on the manganese band was not observed.

If we focus on the bands corresponding to Mn, it is obvious that they consisted of multiple peaks. According to [20,21,24,54], individual bands with different wavelengths can be attributed to Mn^2+^ luminescence with different coordinations. Shorter wavelengths are often associated with tetrahedral coordination of the ion, whereas longer wavelengths indicate octahedral coordination of the Mn^2+^ ion. For this reason, a deconvolution of the luminescence spectra in the region of 500–660 nm was performed. In this case, the maxima of the bands were not fixed. The area ratios, evaluated by the above simulation, are shown in Table 4. The results showed a higher content of octahedrally coordinated Mn^2+^ ions in the glasses containing magnesium. Conversely, in the glasses with barium, this ratio of octahedrally to tetrahedrally coordinated Mn was approximately 1:2.

## 4. Discussion

In the structure of borosilicate glasses, both cerium and manganese serve as modifiers. Cerium in the oxidation states (III+) and (IV+) is likely to exhibit low C_1_- and C_2v_-type symmetry due to its large ionic radius. Consequently, it binds to non-bonding oxides. Manganese in the (II+) and (III+) oxidation states (the (IV+) state has also been observed) is very likely to be surrounded by four oxygens (T_d_ symmetry) or more rarely by six oxygens (O_h_ symmetry) [24,31]. The above oxidation states of both ions have been confirmed through a combination of XPS and EPR methods in studies of crystalline and glass-ceramic materials [24,45,46,47,48,55]. In addition, the crystal field can be estimated from the shape and shifts of the luminescence bands (particularly for manganese). Both of these crystal fields have been confirmed experimentally [21,24,56]. Similarly, the glass-network structure also has an effect, as the luminescence of both elements—especially manganese—is significantly more intense in pure borate glasses than in silicate glasses [51]. This is attributable to the larger number of non-bonding oxygens and consequently low phonon energy.

The spectroscopic measurements demonstrated the presence of the sensitizer–activator pair Ce^3+^ and Mn^2+^ in our glasses, with the concentration of Mn^2+^ ions playing a crucial role in their proper functioning. According to [31], the emission of Mn^2+^ is significantly enhanced by the presence of Ce^3+^ ions compared to the direct excitation of Mn^2+^. As a consequence, the emission of Ce^3+^ decreases with increasing Mn^2+^ concentration. This effect was observed in the prepared glasses, where it was determined that the concentration of MnO should not exceed 1 mol.%. A similar value was also identified as suitable in [37].

According to [57], the equally important redox balance in our glasses was modified by the addition of reducing tin ions. Tin in the form of SnO was added to the glasses as the reducing ion Sn^2+^ in order to shift the redox balance of both ions to lower oxidation states. The experiments revealed that this adjustment significantly shifted the balance between Ce^4+^ and Ce^3+^ in favor of Ce^3+^ while reducing the number of Mn^4+^ and Mn^3+^ ions in favor of Mn^2+^ (as had been assumed). This shift in equilibrium primarily affected the luminescence in the MgO-containing glass, causing a red shift in the luminescence band in the region of 540–640 nm (which corresponds to a change in the coordination of Mn^2+^ from tetrahedral to octahedral) alongside an increase in the luminescence-band intensity. It is important to note that, in addition to the Sn^2+^ ion, the glasses also contained the Sn^4+^ ion. Although the data on the luminescence of both ions are relatively sparse [58,59], they suggest that both ions may also exhibit luminescence bands in glasses corresponding to s–p transitions. For Sn^2+^, the ^3^P_1_ → ^1^S_0_ transition (emission at 400 nm) has been observed. The S_1_ → S_0_ and S_2_ → S_0_ transitions (producing broad yellow–orange emissions at 590–650 nm) recorded for Sn^4+^ can be excited in the UV region (270 or 380 nm) [58,60]. Since our glasses did not contain different tin contents nor did we investigate the oxidation states of this ion in detail, it remains an open question whether and how these two tin ions might influence the luminescence of cerium and manganese.

Optical basicity and its impact on the luminescence spectra of cerium and manganese have been investigated in various types of glass with differing boron or phosphorus contents [11,30,31,32,61,62]. More recently, silicate glass with a very high basicity (0.71) has also been utilized. Because of its poorly cross-linked skeleton, this type of glass allows ions to stabilize their high oxidation states [24]. In our experiments, we used glass with relatively common optical-basicity values, which were systematically varied. The results align well with the abovementioned theory, demonstrating that in the glasses with the lowest optical-basicity values, the desired low oxidation states of Ce and Mn were successfully stabilized. This stabilization, in turn, enhanced the luminescence of both ions in the magnesium-containing silicate glass.

## 5. Conclusions

It is evident from the results that the XPS method is well-suited for monitoring the increase in Ce^3+^-ion concentrations, which is in good agreement with the results from the luminescence spectroscopy at the excitation wavelength of 320 nm. To monitor the Mn^2+^ content, it is appropriate to use EPR methods (where the signals of Mn^3+^ and Mn^4+^ ions can also be detected) and absorption spectroscopy with the characteristic Mn^3+^ band. Three key factors can be utilized to influence the oxidation states of cerium and manganese within the silicate matrix, thereby achieving an optimal Ce^3+^/Mn^2+^ ratio and, consequently, a high luminescence intensity: (i) The first factor is a decrease in the MnO concentration in the glass matrix, which increases the intensity of the Ce^3+^-band maximum in the region of 350–450 nm, corresponding to the d^5^→^2^F_7/2_ and d^5^→^2^F_7/2_ transitions, while also causing its red shift. For a suitable combination of both ions in the glass, the MnO concentration should not to exceed 1 mol.%. (ii) The second possibility is the addition of SnO to the glass. This reduces both ions to low oxidation states (i.e., Ce^4+^ → Ce^3+^ and Mn^x+^ → Mn^2+^) and enhances the luminescence intensity in the region of 540–640 nm, which corresponds to the ^4^T1(^4^G) → ^6^A1(^6^S) transition of Mn^2+^ ions in tetrahedral and octahedral coordinations. (iii) The third option is to adjust the optical basicity of the prepared glasses, ranging from 0.56 to 0.63. This affects the position and intensity of both Ce^3+^ and Mn^2+^ bands. A decrease in optical basicity leads to an increase in the intensity of both bands. This may be achieved through the addition of MgO to the silicate matrix. These findings can be applied to improve the luminescence properties of silicate glasses, which can be utilized in the production of luminescent and radioluminescent optical fibers.

## Figures and Tables

**Table 1:** Compositions (mol.%) of the prepared glasses.

Glass	Photo	Na_2_O	MgO	CaO	BaO	SiO_2_	B_2_O_3_	Al_2_O_3_	MnO	CeO_2_	SnO
Ba–Mn reference	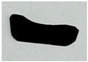	14.94	-	-	7.97	54.49	17.55	3.05	1.99	-	-
Ba2%Mn	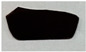	15.23	-	-	8.12	54.97	15.53	3.11	2.03	1.01	-
Ca2%Mn		15.23	-	8.12	-	54.97	15.53	3.11	2.03	1.01	-
Mg2%Mn		15.22	8.12	-	-	54.94	15.52	3.11	2.03	1.06	-
Ba1%Mn	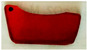	15.25	-	-	8.13	55.93	15.56	3.12	1.00	1.01	-
Ca1%Mn		14.89	-	7.94	-	54.61	17.49	3.05	0.98	1.04	-
Mg1%Mn		14.89	7.94	-	-	54.61	17.49	3.05	0.98	1.04	-
Ba1%MnSn	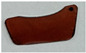	15.25	-	-	8.13	54.93	15.56	3.12	1.00	1.01	1.00
Ca1%MnSn		15.26	-	8.13	-	54.96	15.56	3.12	1.00	0.97	1.00
Mg1%MnSn		15.25	8.13	-	-	54.93	15.56	3.12	1.00	1.01	1.00
Ba0.2%Mn	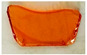	15.18	-	-	8.09	56.94	15.48	3.10	0.20	1.01	-
Ca0.2%Mn		15.17	-	8.10	-	56.94	15.48	3.10	0.20	1.01	-
Mg0.2%Mn		15.17	8.09	-	-	56.94	15.48	3.10	0.20	1.01	-
Ba–Ce reference	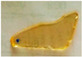	15.17	-	-	8.09	57.16	15.47	3.10	-	1.01	-

**Table 2:** General properties of the prepared glasses.

Glass	*ρ* (g·cm^−3^)	*n*_int_ (589 nm)	*N* (632.8 nm)	A	B	C/10^8^	Λ_th_	Λ
Ba–Mn standard	2.89 ± 0.06	1.5570	1.5542	1.5355	9751.7722	−7.9915	0.590	0.63
Ba2%Mn	2.87 ± 0.11	1.5609	1.5581	1.5391	9499.3330	−6.5425	0.608	0.62
Ca2%Mn	2.48 ± 0.07	1.5440	1.5411	1.5231	9292.6726	−7.1317	0.584	0.63
Mg2%Mn	2.54 ± 0.07	1.5292	1.5264	1.5088	9098.0695	−7.0272	0.577	0.57
Ba1%Mn	2.89 ± 0.06	1.5570	1.5542	1.5355	9751.7722	−7.9915	0.600	0.62
Ca1%Mn	2.56 ± 0.04	1.5420	1.5392	1.5214	9299.9315	−7.4192	0.577	0.57
Mg1%Mn	2.49 ± 0.03	1.5250	1.5222	1.5043	9342.0628	−7.5881	0.566	0.56
Ba0.2%Mn	2.91 ± 0.05	1.5555	1.5527	1.5344	9427.5046	−7.2049	0.605	0.60
Ca0.2%Mn	2.51 ± 0.06	1.5401	1.5373	1.5197	9115.0583	−6.9926	0.588	0.61
Mg0.2%Mn	2.48 ± 0.02	1.5226	1.5202	1.5024	9316.2869	−7.7113	0.573	0.57
Ba1%MnSn	2.91 ± 0.06	1.5609	1.5581	1.5403	9041.5247	−6.5746	0.605	0.61
Ca1%MnSn	2.66 ± 0.04	1.5450	1.5421	1.5242	9301.7890	−7.1641	0.596	0.58
Mg1%MnSn	2.55 ± 0.03	1.5280	1.5252	1.5080	8882.9632	−6.7283	0.580	0.58
Ba–Ce standard	2.84 ± 0.03	1.5545	1.5517	1.5335	9394.3291	−7.2784	0.599	0.63

**Table 3:** The areas of the peaks obtained by deconvolution of the absorption spectra.

Glass	Mn^4+^ Band 469 nm	Mn^3+^ Band 500 nm
Ba0.2%Mn	0.054	3.294
Ba1%Mn	3.597	28.371
Ba2%Mn	4.301	119.155
Ba-Mn standard	9.123	348.300
Mg1%Mn	3.718	26.859
Ca1%Mn	5.977	24.553
Ba1%Mn	3.597	28.371
Mg1%MnSn	0.075	17.592
Ca1%MnSn	1.001	20.905
Ba1%MnSn	1.079	22.958

**Table 4:** The characteristics of the Mn^2+^ peaks obtained by deconvolution of the luminescence spectra.

Glass	Mn^2+^ Band Tetrahedral		Mn^2+^ Band Octahedral	
	Position nm	Area/10^6^	Position nm	Area/10^6^
Ba0.2%Mn	-	-	-	-
Ba1%Mn	540	22.702	601	10.247
Ba2%Mn	540	6.628	597	8.156
Ba-Mn standard	-	-	-	-
Mg1%Mn	551	28.003	598	54.165
Ca1%Mn	538	12.362	590	42.464
Ba1%Mn	540	22.702	601	10.247
Mg1%MnSn	539	9.478	583	63.439
Ca1%MnSn	539	26.365	593	7.256
Ba1%MnSn	531	19.762	602	11.118

## Data Availability

The data presented in this study are available from the corresponding author upon reasonable request.
